# Supplementary material for: Near-Infrared Light-Triggered Nitric Oxide Nanogenerators for NO-Photothermal Synergistic Cancer Therapy
Source: Nanomaterials (Basel). 2022 Apr 14;12(8):1348. doi: 10.3390/nano12081348 (PMC9029494; doi:10.3390/nano12081348)
Supplement: Supplementary file 1 [file nanomaterials-12-01348-s001.zip › nanomaterials-1666101-supplementary.pdf]

nanomaterials

Article

Near-Infrared Light-Triggered Nitric Oxide Nanogenerators for NO-Photothermal Synergistic Cancer Therapy

Weiwei Liu [1,2], Farouk Semcheddine [1], Zengchao Guo [1], Hui Jiang [1,*] and Xuemei Wang [1,*]

[1] State Key Laboratory of Bioelectronics, National Demonstration Center for Experimental Biomedical Engineering Education, School of Biological Science and Medical Engineering, Southeast University, Nanjing 210096, China; liuw182@163.com (W.L.); fou_semch@yahoo.fr (F.S.); rmcqh242526@163.com (Z.G.)
[2] School of Chemistry and Chemical Engineering, Southeast University, Nanjing 210096, China
* Correspondence: sungi@seu.edu.cn (H.J.); xuewang@seu.edu.cn (X.W.)

Supplementary Figures

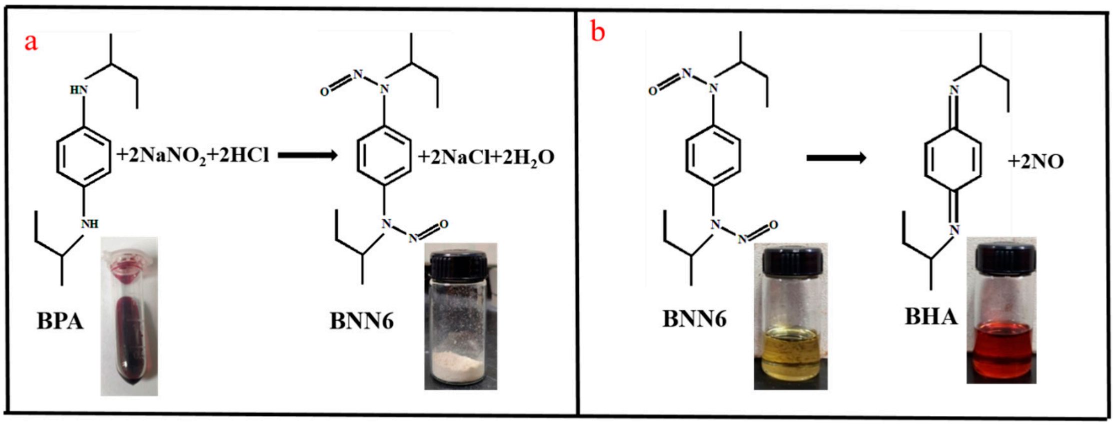

Figure S1. (**a**) The synthesis route of BNN6 and the change from red liquid to beige solid in the reaction process (inset digital pictures); (**b**) The decomposition route of BNN6 and the color change during decomposition (inset digital pictures).

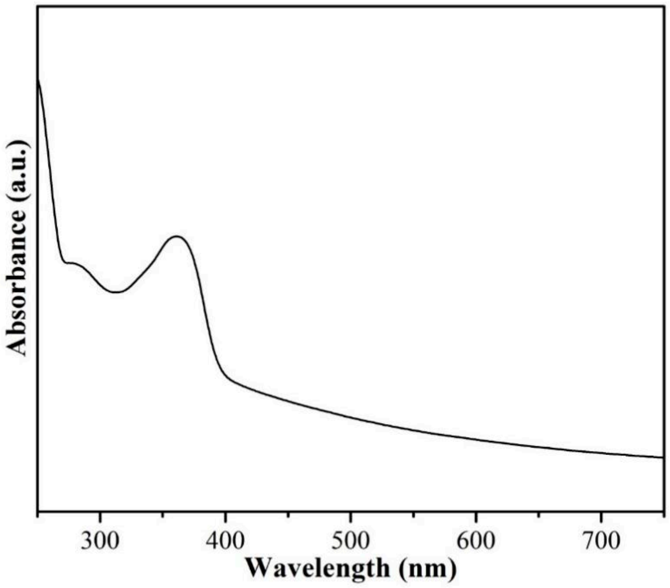

Figure S2. UV-vis absorption spectra of BNN6.

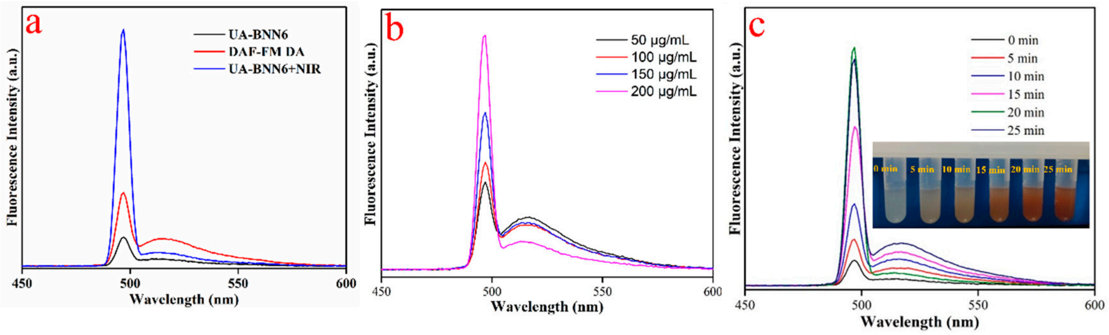

Figure S3. (**a**)Fluorescence curve of DAF-FM DA, of UA-BNN6 and of UA-BNN6+NIR. (**b**) Fluorescence curves of PBS solutions of UA-BNN6 with different concentrations after irradiation by NIR (808 nm, 1.0 W cm^{-2}). (**c**) Fluorescence curve of UA-BNN6 PBS solution (200 μg/mL) after by irradiation NIR for different time. Inset: the color change during decomposition.

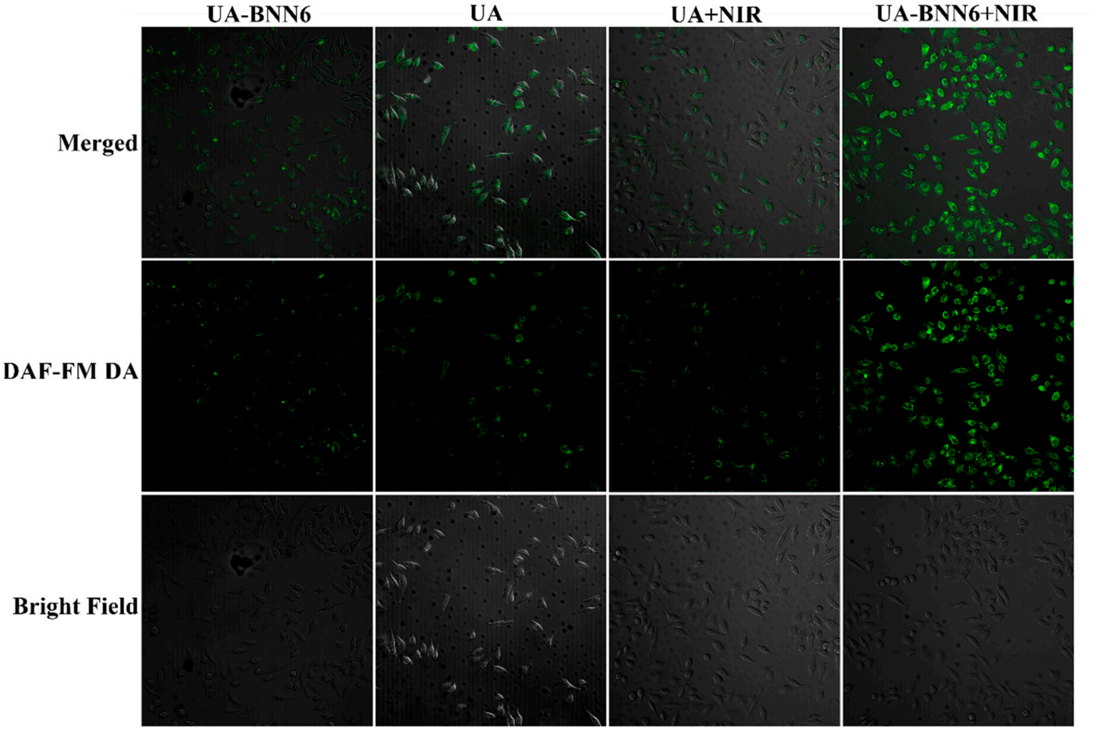

Figure S4. Confocal images of intracellular generation of NO in HeLa cells after treatment with UA, UA-BNN6, UA+NIR and UA-BNN6+NIR.

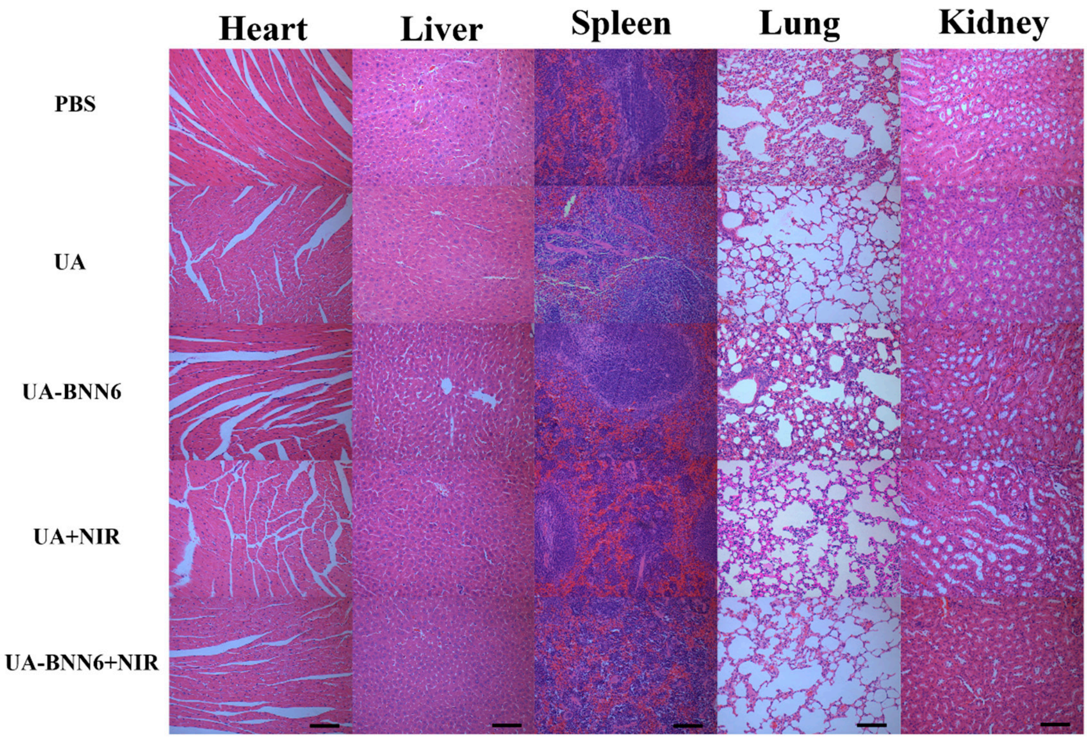

Figure S5. H&E staining of tissue sections of main organs and tumors in nude mice (scale bars 100 μm).
